# What drives bank liquidity creation? The interaction of monetary policy and strategic scope

**DOI:** 10.1016/j.heliyon.2024.e24131

**Published:** 2024-01-09

**Authors:** Japan Huynh

**Affiliations:** Faculty of Finance and Banking, Ho Chi Minh City Open University, 35-37 Ho Hao Hon, Co Giang Ward, District 1, Ho Chi Minh City, 700000, Viet Nam

**Keywords:** Bank liquidity creation, Interest rates, Monetary policy, Money supply, Strategic scope

## Abstract

The research investigates how the marginal influence of monetary policy on the expansion of liquidity creation changes depending on the strategic scope of banks. Through a sample of Vietnamese commercial banks between 2007 and 2019, we estimate measures of liquidity creation that consider all banking items and build up various monetary policy indicators. Empirical regressions are achieved with the dynamic generalized method of moments (GMM) estimator that is fitted to tackle the endogeneity issue. The study confirms the existence of the bank liquidity creation channel, i.e., an easing monetary policy boosts the banks' liquidity creation ability, both when the central bank reduces interest rates and injects more capital into the market. For banks with a strategic scope that relies more on non-interest revenues or diversifies more into various banking segments, the relationship between monetary policy and the generation of bank liquidity is strengthened when the central bank adjusts interest rates but mitigated if it changes the money supply using quantitative-based monetary tools. This paper expands the literature stream on monetary policy's liquidity creation channel, which is essential to the economy but has been little explored, by shedding light on the moderating role of bank income. Additionally, this study employs many policy indicators based on interest- and non-interest-rate tools to avoid misleading conclusions on the policy stance.

## 1 Introduction

The critical role of banks is to provide the economy with liquidity [1]. In this vein, liquidity creation is created when banks use liquid liabilities to fund illiquid assets (on balance sheets) and through loan commitments (off balance sheets) [2]. It is believed that bank liquidity production would greatly influence the financial sector as well as the entire economy. On one side, liquidity creation might effectively fund economic expansion [3,4]. On the other side, liquidity creation may adequately predict banking collapses and financial crises [5]. Due to the remarkably indispensable role of liquidity creation, abundant research has been done recently to examine its determinants, especially since Berger and Bouwman [6] invented a novel and comprehensive standard to gauge liquidity creation.^1^

Recent research by Berger and Bouwman [7] examines the reaction of bank liquidity creation to fluctuations in monetary policy and suggest the so-called “bank liquidity creation channel”, which builds on the well-positioned bank lending channel in the existing literature. If the bank lending channel works when monetary policy alters loan supply due to affected loanable funds [8], the bank liquidity creation channel theoretically operates in a more complicated and comprehensive route. Berger and Bouwman [7] indicate that monetary policy could widely influence all banking activities on- and off-balance sheets in the sense that an expansionary monetary policy should enhance the volume of liquidity created.

Thus far, the understanding of how monetary policy shapes liquidity creation is quite limited. In the seminal paper by Berger and Bouwman [7] mentioned above, they witness the liquidity creation channel in the US. However, their evidence is rather weak and mixed: the effect of changes in monetary policy on liquidity creation is statistically significant but economically diminutive at small banks, while ambiguous patterns are found at medium and large banks. Furthermore, the authors only adopt the federal funds rate to develop their monetary policy measures, which perhaps restrict the operation of the liquidity creation channel via different mechanisms with different monetary tools. In subsequent studies that desire to confirm and develop the findings from Berger and Bouwman [7], Dang and Dang [9] and Pham et al. [10] investigate multiple bank-specific factors (e.g., capital buffers, bank size, or bank risk) as the moderators of the liquidity creation channel. Nevertheless, using these standard characteristics is insufficient to discuss the causal impacts of monetary policy since they cannot provide an adequate evaluation of the motivation, willingness, and capability of banks to adjust banks’ output [11]. Recently, Dang and Huynh [12] overcome this shortcoming by shedding light on the moderating effect of financing structure and pricing power on how monetary policy shapes the production of liquidity.

This study complements the literature by analyzing the conditioning role of non-interest income on the association between bank liquidity creation channel and monetary policy. Based on the well-discussed literature on bank income models, we claim that this aspect needs to be carefully considered, rather than other bank-level features, when exploring the working of the liquidity creation channel. There has been limited evidence on the link between the bank lending channel and non-interest income that has inspired this research (see section 2 for a review). From such indication, we can expect that banks diversifying from traditional lending activities into non-traditional banking segments are less concerned about the monetary policy implementation that rules the bank liquidity creation channel, thereby weakening its potency. However, this mechanism may not hold for the manner monetary policy drives liquidity creation – regarded superior to bank lending in exhibiting bank output as it covers all banking components like loans, funding, and even guarantees [6,13]. Moreover, in multiple situations, central banks employ diverse policy instruments with distinct operating processes and complicated outcomes [14], thus potentially complicating the interdependence of monetary policy, liquidity creation, and bank income models. Overall, examining the marginal impacts of strategic scope on the liquidity creation channel remains an relevant empirical topic and is worth an in-depth analysis.

To conduct the estimations, we utilize the metrics proposed by Berger and Bouwman [6], in which we estimate two measures of total and on-balance-sheet liquidity creation. As a striking feature of this study, we construct multiple monetary policy measures based on the central bank's interest- and quantitative-based tools. Concretely, these measures include short-term lending rates, policy rates, purchases of treasury bills on the open market, and the reserves of foreign exchange. Banks' strategic scope is captured in terms of the non-interest income share to indicate the shift towards non-interest banking segments and the diversification indexes to illustrate the income diversification across conventional and non-conventional activities, as well as across all banking activities. Empirical regressions are achieved with the dynamic generalized method of moments (GMM) estimator that is fitted to tackle the endogeneity issue.

Our analysis is performed for Vietnam, a market with essential conditions that make it a favorable case study for the marginal effects of bank income models on the monetary transmission via the liquidity creation channel. First, Vietnam owns a relatively underdeveloped capital market, so bank output is recognized as a core force in driving economic growth. This fact may reinforce the transmission of monetary policy via banking channels. Second, as the sole monetary authority, the State Bank of Vietnam (SBV) may establish various objectives, including inflation control, productivity expansion, and stable macroeconomics [15]. In this regard, The SBV commonly mixes interest- and quantitative-reliant policy instruments to fulfill its purpose. Third, income diversification strategies have constantly been taking place in the Vietnamese banking sector. Accordingly, the income structure of banks has changed significantly, associated with many essential reforms in banking regulations. However, the bright and dark sides of this shift have still been left open and not analyzed carefully by regulators and academics.

With these designs, our study offers some contributions. It expands the literature line on the bank liquidity creation channel, which is of importance to the economy but has been little explored so far. Specifically, clarifying how the marginal influence of monetary policy on the expansion of liquidity creation changes depending on the strategic scope of banks is a key innovation of this paper. Unlike the standard bank-level factors, income models may reveal more about the ability, incentive, and willingness of banks to modify their core production. Additionally, this study employs various monetary policy indicators, based on both interest- and non-interest-based tools, to conduct the analysis. This contribution indicates that the use of different monetary measures supports the insightful argument that the implications regarding the moderating role of strategic scope could be misleading if based on a single monetary indicator. Generally, our empirical analysis should produce not only novel conclusions on the linkage between strategic scope and the liquidity creation channel but also lend unique perspectives to monetary authorities in Vietnam and similar emerging economies, where the effects of income diversification on the potency of monetary transmission should have garnered much greater attention due to the rising diversity strategies over the past years.

## 2 Literature review

The study is related to two key literature segments. The first one studies the importance of strategic diversification, and the second one includes the banking channel of monetary transmission. From these theoretical and empirical documents, the potential mechanisms to explain how liquidity creation responds to monetary shocks varying according to bank income models will be developed.

The non-interest income literature has mainly discussed the benefits and costs as banks diversify into non-traditional business lines. When it comes to the upsides of non-interest income, diversified banks could obtain more confidential data on client quality and access to a larger pool of possible loans [16]. This route alleviates informational asymmetries, improves credit supervision, and expands the network of prospective customers. However, non-lending segments may have several downsides. First, the majority of these operations are short-term by nature and have lower switching costs than conventional banking categories [17]. This motivates the higher volatility of non-interest activities. Second, expanding into non-interest business lines may raise the degree of financial leverage since banks are not obliged by authorities to carry equity capital against these lines [18]. Third, heavier reliance on non-interest activities could hamper bank lending as less exposure to credit activities may motivate bank management to be less cautious in loan-granting divisions [19].

Many empirical studies have been conducted to examine the impact of non-interest revenue on the functioning of the banking system. Commonly considered aspects include financial efficiency, risk-return tradeoff, bank valuation, and financial stability. In general, these studies show mixed evidence. For a careful review of the related empirical literature strand on the impact of non-interest income on various financial indicators of banks, see Williams and Rajaguru [20] and Addai et al. [21].

Concerning academic documents on the liquidity creation channel, we should first discuss how it works in detail. On balance sheets, an expansion of monetary policy is suggested to promote liquidity creation through increased loans (as commonly witnessed in the bank lending channel) and deposits. Relaxing monetary policy may increase the loanable funds available and decrease the funding costs by substituting higher-cost funding sources with cheaper deposits [22]. Accordingly, banks would react by granting more loans, including those to the applicants that may otherwise be rationed [23]. Off balance sheets, banks may grant more commitments due to the availability of loanable funds and the drop in these funds’ costs after an expansionary monetary policy [24]. This manner is believed to increase off-balance-sheet liquidity creation.

Though Berger and Bouwman [7] introduce the presence of the bank liquidity creation channel in their pioneering paper, with limited evidence indicating the minor impact at small banks and ambiguous patterns at medium and large banks, they still make the final conclusion that monetary policy appears not to be a powerful tool to drive banks' core function. Developing this novel research path, some scholars explore the conditioning effects caused by bank-level variables on how liquidity creation responds to monetary adjustments [9,10,12]. The authors provide more convincing evidence to certify the existence of the bank's mechanism for creating liquidity and furthermore, they reveal that this banking channel operates differently across banks with different bank-specific conditions. In fact, their work opens a critical research avenue on the conditions to operate the liquidity creation channel.

Our current research topic also builds on the literature analyzing the bank lending channel of monetary policy, which is only a constituent part of the bank liquidity creation channel. While the empirical evidence on the liquidity creation-monetary policy linkage is thin, the available literature on the nexus between monetary policy and bank lending is vibrant. For the past decades, there has been considerable interest of policymakers and academia in exploring the working of the bank lending channel. In this vein, we observe many papers interested in the way banks react to variations in the monetary policy based on the strengths of their balance sheets, captured by qualities specific to banks, such as bank size, liquid asset holdings, and bank capital. They provide well-known evidence that banks with weaker financial profiles are more difficult to raise external funds in the market, so their credit distribution is more sensitive to monetary policy changes than their counterparts with stronger balance sheets [[25], [26], [27]]. An outstanding review on the working of the bank lending channel moderated by various factors could be discovered in Cheng and Wang [28], Wang et al. [29], and Imbierowicz et al. [30].

Though not directly showing the relationship between activity strategies and the impact of monetary policy on bank liquidity creation, existing research documents, both theoretical and empirical, have contributed to suggesting some potential mechanisms for this relationship. Many works indicate that banks’ liquidity creation function decreases with more reliance on non-conventional activities or a higher degree of bank revenue diversification in both advanced and developing markets [31,32]. The scholars argue that: (i) a wider span of business models consumes numerous banking resources, causing banks to fail to better meet the liquidity demand of customers, and (ii) the funds of non-interest-based banks could be allocated to many different asset categories instead of only loans. Hence, these mechanisms indicate that strategic scope relying more on non-interest sources could curb the transmission of monetary policy.

In sharp contrast, the former literature has generated competing reasons why non-interest income strengthens the liquidity creation channel. First, due to lower switching costs of non-lending-based banks [17], bank activities tend to fluctuate to a larger extent amid monetary policy shocks. For example, in the event of an adjusted interest rate framework, banks with less enduring ties with borrowers are less likely to manage their asset portfolios and thus make them more volatile. Second, since banks are not obligated by regulators to boost capital against the growth of non-interest operations [18], institutions having more reliance on non-interest activities could leverage more freely than those preferring lending lines. This makes banks more flexible to modify their financial leverage amid monetary policy shocks, resulting in more variation in the bank liquidity creation. Third, banks usually play the part of key partners with central banks in open market operations and foreign exchange intervention [33]. In this regard, it should be stressed that non-interest-based banks would be more interested in trading financial assets and foreign currencies with central banks, thus exaggerating the banks’ ability to produce liquidity when central banks inject funds.

Despite the significance of the relationship between monetary policy and liquidity creation, this channel is not well understood. Only a few authors have used bank characteristics to discuss how monetary policy influences liquidity production in this regard. Nevertheless, there has been no evidence of the link between the bank liquidity creation channel and bank business models. This motivates us to fill the present gap in the literature.

## 3 Data and methodology

### 3.1 Data

The paper collects bank-level data from the financial reports of commercial banks in Vietnam from 2007 to 2019. We omit banks that have been obligatorily obtained or placed under exceptional control by the SBV due to their radically distinct commercial orientations (four banks by the end of 2019). We also disregard foreign-owned and joint-venture banks due to their small size and minor role within the Vietnamese banking system (two joint-venture and nine foreign-owned banks at the end of 2019). Importantly, these removed institutions do not disseminate the information necessary to calculate bank liquidity creation, a central aspect of this research. We exclude banks without at least five consecutive years of financial reporting.^2^ Our filtering process ultimately leads to an unbalanced panel data sample, consisting of 31 Vietnamese commercial banks, with a total of 362 observations, making up more than 90% of the banking system's total assets in Vietnam. These bank-level data from financial reports are publicly available on the official websites of each bank.

Regarding the country-level data, the short-term lending rates, foreign exchange reserves, and the SBV's claims on government are sourced from the database of the International Financial Statistics website, while the policy rates come from the SBV's website. In addition, we gather stock market information from the Vietstock website and economic growth and inflation indicators from the website of World Development Indicators. Before conducting our research, we winsorize all bank-level variables at the 2.5 and 97.5 percentiles to minimize the influence of extreme outliers.

### 3.2 Model

To examine the conditioning roles of non-interest income in the liquidity creation channel, we build on the previous works of Berger and Bouwman [7] and specify the model as follows:

(1) $${LC}_{i,t\;}={\;\alpha }_{0\;}+{\;\alpha }_{1}\times {LC}_{i,t–1\;}+{\;\alpha }_{2}\times {MP}_{t–1\;}+{\;\alpha }_{3}\times {MP}_{t–1}\times {BSS}_{i,t–1\;}+{\;\alpha }_{4}\times {BSS}_{i,t–1\;}+{\;\alpha }_{5}\times X_{i,t–1\;}+{\;\alpha }_{6}\times Z_{t–1\;}+u_{i,t\;}$$

where i denotes banks and t captures years. LC is the dependent variable, measured by the annual percentage change in bank liquidity creation. We also added the lagged dependent variable to highlight the dynamic nature of liquidity creation. MP is the monetary policy variable. Banks' strategic scope (BSS) is captured either by the non-interest income share or the diversification index between interest- and non-interest sources. X contains bank-level factors and Z is a vector of macroeconomic controls. $u_{i,t\;}$ reflects the error term. We employ one-year lags of independent variables in equation [1] because, consistent with the former literature, banks may not respond immediately to monetary variations [34] and we also desire to curtail the potential endogeneity bias. The interaction term MP×BSS is our main interest, which indicates the heterogeneity in the liquidity creation channel according to banks' strategic scope.

Our dynamic panel model is regressed using the two-step system GMM setting [35]. This econometric approach could handle the endogeneity problem by employing lagged regressors as instruments. Following the suggestion of Roodman [36], we limit the proliferation of internally created instruments to wipe out the issue of “too many instruments”. We then conduct the Hansen test to jointly validate the set of instruments used. We also perform the Arellano-Bond test to diagnose the unavailability of the second-order autocorrelation in idiosyncratic residuals.

### 3.3 Variables

#### 3.3.1 Bank liquidity creation measures

We adopt the three-stage method of Berger and Bouwman [6] to define liquidity creation measures for commercial banks. For the first stage, we classify all banking items on- and off-balance sheets into liquid, semiliquid, or illiquid groups. It is determined by the ease, cost, and time it takes clients to acquire liquidity from banks, as well as the ease and cost of banks' commitments to satisfy these liquidity demands. On the asset side, for instance, the holdings of additional loans (or securities) boost (or reduce) bank liquidity creation. On the liability side, possessing more liquid deposits (or illiquid equity) results in increased (decreased) liquidity. Off-balance-sheet items are handled in an identical manner as assets. In the second stage, we assign each classified group the corresponding weights of +1/2, 0, or −1/2 based on the general mechanism that both financing activities and asset distribution contribute equally to the generation of bank liquidity. Accordingly, one unit of liquidity is produced if one unit of illiquid assets is funded by one unit of liquid liabilities (for instance, banks use customer deposits to provide loans), and one unit of liquidity is wiped out when one unit of liquid assets is covered by one unit of illiquid liabilities or equity (for example, banks use equity to purchase securities). In other words, the scale of this weight is +1/2 and −1/2, based on the supposition that only half of the liquidity generated may be attributed to the origins and usages of funds. The weight of 0 is ascribed to assets and liabilities that are semi-liquid. In addition, the weight for the off-balance sheet account is determined using the same criterion as the on-balance items. Finally, in the third stage, we reach liquidity measures by incorporating the outcomes from the first two steps into the equation as follows:

(2) $$\begin{array}{c} Liquidity\;creation=1/2\times \left(Illiquid\;assets+Liquid\;liabilities+Illiquid\;guarantees\right)\\ 0\times \left(semiliquid\;assets+semiliquid\;liabilities+semiliquid\;guarantees\right)\\ –1/2\times \left(Liquid\;assets+Illiquid\;liabilities\;and\;equity+Liquid\;guarantees\right) \end{array}$$

Berger and Bouwman [6] propose a total of four liquidity measures, which are determined by whether the classification procedure is depending on maturity or category (“mat” or “cat”, respectively) and excluding or including off-balance-sheet components (“nonfat” or “fat”, respectively). This study utilizes the “cat fat” displayed in equation [2], which is a metric that organizes loans by category and incorporates off-balance-sheet items, and the “cat nonfat” liquidity creation version, which is obtained by classifying loans based on category and dropping off-balance-sheet items. Such a selection is because, on the one hand, these procedures are favored by Berger and Bouwman [6] and, on the other hand, our dataset cannot afford detailed information on the item maturity. Table 1 presents the categories and weights of banking activities for liquidity creation calculation in this study. It should be noted that in the context of Vietnam, our specific procedure is adopted from the original metrics of Berger and Bouwman [6] and also the adjusted versions of Dang and Huynh [12] to generate more effective liquidity creation measures. Regarding the sources of data to calculate liquidity creation, we can access the balance sheets of commercial banks for the construction of the “cat nonfat” liquidity creation version. Additionally, we further need the notes to financial statements for detailed data items to compute the “cat fat” measure, which requires the information on the off-balance-sheet items.

**Table 1:** Categories and weights of banking activities for liquidity creation calculation.

Illiquid assets (+1/2)	Semiliquid assets (0)	Liquid assets (−1/2)
Corporate loans Consumer loans Other assets	Interbank loans	Cash and due from other banks Securities
**Liquid liabilities (** + **1/2)**	**Semiliquid liabilities (0)**	**Illiquid liabilities and equity (–1/2)**
Customer deposits Trading liabilities	Deposits from other banks Other borrowed funds	Other liabilities Equity capital
**Illiquid guarantees (** + **1/2)**	**Semiliquid guarantees (0)**	**Liquid guarantees (–1/2)**
Loan commitments Loan guarantees Letters of credit	Other guarantees	Derivatives contracts

#### 3.3.2 Monetary policy indicators

As a unique feature of this study, we employ various monetary policy indicators to examine the bank liquidity creation channel. Following a common practice exhibited in early works and some papers that focus on implementing Vietnam's monetary policy, we approach interest- and non-interest-rate tools [15]. The former includes short-term lending rates and policy rates, while the latter contains the SBV's securities trading and the reserves of foreign exchange. The rationales of selecting these indicators and how they should work in the estimation stage are clarified as follows.

Lending rates could be seen as a highly effective monetary policy indicator for Vietnam. The SBV usually executes administrative orders and announces regulations on interest rate ceilings to set up a framework of lending rates in the market. Besides, the SBV's various efforts tend to converge on targeted lending rates which may fully reflect the SBV's monetary policy stance. As the lender of last resort, the SBV also regularly uses policy rates in fund transactions with the commercial banking system. Accordingly, refinancing rates are assigned to short-term loans, and rediscounting rates are given for valued paper discounts. The study considers the level values of all three forms of interest rates for the analysis, in which their higher values indicate a contractionary monetary policy and their lower values signify an expansionary monetary policy.

Along with the crucial interest tools, the SBV actively engages in open markets to modify the market's money supply by acquiring and transferring financial assets with institutions. This quantitative-based tool is considered as a key tool of monetary policy in Vietnam in recent years, in the context that monetary tools such as required reserves and basic interest rates have almost been fixed for long periods. In line with the literature in proxying this tool, we take the SBV's claims on government, and then we use its natural logarithm form in the regression analysis [37]. Contrary to interest-rate measures, the greater values of this indicator reflect an expansionary policy.

Another interesting tool that the SBV could use to alter the domestic money supply is through the foreign exchange intervention. Unless completely sterilized, this action may potentially adjust the monetary base and induce a power similar to monetary policy. This supplementary tool has shown its important position in recent times when the SBV has consistently highlighted the accumulation of foreign exchange resources. To effectively capture the working of foreign exchange intervention, we inherit the formula of Chen et al. [38], which is written as follows:

(3) $$Foreign\;exchange\;reserves=\left(\frac{fxr}{GDP}\right)\times \frac{\sigma \left(fxr\right)}{\sigma \left(fxr\right)+\sigma \left(eer\right)\;}$$

where (fxr/GDP) is the foreign exchange holdings as a share of GDP, and the modifier, σ(fxr)/[σ(fxr)+σ(eer)], reflects the adjusted component to feature the exchange regime. In some detail, σ(fxr) and σ(eer) are the standard deviations of foreign exchange holdings and real effective exchange rates, respectively. It is presumed that a country will intervene in the foreign exchange market by buying or selling its reserves of foreign exchange. A rise (fall) in foreign exchange holdings is interpreted as a potential growth (decrease) in domestic market liquidity. However, the degree to which fluctuations in foreign reserves can be attributable to foreign exchange interference depends on whether the country's exchange rate system is floating or fixed. Consequently, in equation [3] we modify (fxr/GDP) with the modifier σ(fxr)/[σ(fxr)+σ(eer). If the exchange rate is entirely floating, σ(fxr) and σ(fxr)/[σ(fxr)+σ(eer) should equal 0.^3^ Alternatively, if the exchange rate is completely fixed, σ(eer) is anticipated to equal 0 and the ratio is therefore equal to 1.^4^ As the ratio σ(fxr)/[σ(fxr)+σ(eer) approaches 1, a more significant variation in foreign reserves is expected to be connected to more active foreign exchange interventions. With this setting, an increase (decrease) of foreign exchange reserves is translated into a potential expansion (contraction) of monetary policy.

Additionally, it is necessary to discuss the working of other monetary policy measures taken in Vietnam. Since 1990, the SBV has utilized reserve requirements to regulate banks' credit supply. This was a potent monetary policy instrument during the early stages of the SBV's operation. However, the position of reserve requirement has been significantly undermined by the SBV's recent adoption of other instruments, including open market operations and policy rates. As a result, the SBV has retained a stable reserve requirement ratio since 2012. In addition, since 2000, the SBV has implemented the base interest rate mechanism to limit bank lending rates. However, since 2010, the SBV has not routinely amended and fixed this kind of interest rate. Therefore, we do not consider these monetary policy tools in the paper.

#### 3.3.3 Strategic scope variables

In line with most studies in the relevant literature [[39], [40], [41], [42]], we display banks’ strategic scope or income models using two key proxies in this study. The first is determined by the percentage of non-interest income to total operating income, which demonstrates a trend toward non-interest banking divisions, and the second one is calculated via the following equation [4] to denote the degree of income source diversification, under the approach of the Herfindahl-Hirschman Index:

(4) $$HHInetvsnon=1–\;\left({\left(noninterest\;income\;proportion\right)}^{2}+{\left(interest\;income\;proportion\right)}^{2}\right)$$

Following the suggestion of Meslier et al. [43], excluding negative revenue sources ensures that the non-interest income ratio falls within the 0 to 100 spectrum and the earnings diversification index lies within the range of 0–1.

#### 3.3.4 Control variables

Our work belongs to an emerging strand of literature on the determinants of liquidity creation. Thus, some critical controls are considered in our paper to better identify the liquidity creation trend in the Vietnamese banking sector.^5^ We include bank size based on the motivation that larger banks may benefit from the scale economies to expand their liquidity aggressively [44]. We control bank capital, which is expected to alter bank liquidity creation in two contrasting ways. Under the “financial fragility” claim, banks with more capital may be more cautious with risky investments, which potentially mitigate their liquidity creation [45] and entirely challenges the “risk absorption” hypothesis [46]. Liquidity position should also be taken into account because we realize that liquid banks are more able to finance their subsequent investments as well as grow their loan supply in the future [47].

Apart from bank-specific characteristics, we also introduce several macroeconomic factors that could potentially drive bank liquidity creation. It is well established in the literature that better macroeconomic conditions cultivate investment opportunities, which may broadly change the extent that banks create liquidity [13,48]. Accordingly, we employ three additional factors: the economic growth, inflation, and stock market, to control the external environment. This inclusion further captures the overall time-variant impacts of the economy that rule bank behavior.

## 4 Results

### 4.1 Preliminary analysis

In Table 2, two liquidity creation measures are distributed with the large standard deviations and the wide ranges from the minimum to the maximum. This observation indicates a considerable fluctuation in the change of liquidity creation across banks in Vietnam. Shifting our focus to monetary policy, we see the substantial standard deviations for each of the five dimensions, indicating the remarkable adjustments on the SBV's monetary policy during the time. Additionally, an interesting result emerges, revealing that interest rates in Vietnam have never touched zero, which is needed to ensure that an asymmetric pattern for monetary policy indicators will not cause biased and inconsistent estimation results.

**Table 2:** Descriptive statistics of all variables.

	Mean	SD	Min	Max	Definitions
*Bank liquidity creation measures*					
Liquidity creation (“cat fat”)	23.035	81.957	−254.174	326.105	Annual percentage change of “cat fat” bank liquidity creation measure (%)
Liquidity creation (“cat nonfat”)	20.545	76.870	−311.378	225.238	Annual percentage change of “cat nonfat” bank liquidity creation measure (%)
*Bank-level variables*					
NIIshare	23.296	14.907	2.063	69.606	Non-interest income/Operating income (%)
HHInetvsnon	0.308	0.125	0.026	0.498	Income diversification measure following the Herfindahl-Hirschman Index
Capital	10.280	5.351	4.384	29.008	Equity capital/Total assets (%)
Liquidity	17.762	10.429	5.090	47.311	Liquid assets/Total assets (%)
Size	31.967	1.294	29.404	34.630	Natural logarithm of total assets
*Monetary policy indicators*					
Lending rates	10.400	3.328	6.960	16.954	Average short-term lending rates (%)
Refinancing rates	8.042	2.547	6.000	15.000	Refinancing rates announced by the SBV (%)
Rediscounting rates	5.894	2.660	3.500	13.000	Rediscounting rates announced by the SBV (%)
Foreign exchange reserves	17.498	6.361	7.632	29.460	Foreign exchange reserves normalized by the GDP and the foreign exchange regime (%)
Central bank claims	31.042	0.813	29.982	32.040	Natural logarithm of the SBV's claims on government
*Macroeconomic variables*					
Inflation	7.495	6.226	0.631	23.115	Annual inflation rate (%)
Economic growth	6.245	0.642	5.247	7.130	Annual percentage change of GDP (%)
Stock return	7.425	29.655	−65.953	56.761	Annual percentage change of VNindex (%)

Table 3 illustrates the matrix of correlation coefficients between pairwise variables. As expected, the correlation coefficients between our monetary policy indicators are relatively large, thereby confirming the argument that the SBV often employs different policy instruments concurrently to achieve its monetary objectives. Another important result that we should be concerned about in this part is the severe multicollinearity problem. In this regard, all independent variables are not excessively correlated with each other, except for the significant correlation between the inflation rate and monetary policy interest rates. Thus, we proceed to the next estimation step of main interest without including the inflation variable in the economic model.^6^

**Table 3:** Correlation coefficients matrix.

	Liquidity creation (“cat fat")	Liquidity creation (“cat nonfat")	NIIshare	HHInetvsnon	Capital	Liquidity	Size	Lending rates	Refinancing rates	Rediscounting rates	Foreign exchange reserves	Central bank claims	Inflation	Economic growth	Stock return
Liquidity creation (“cat fat”)	1.000														
Liquidity creation (“cat nonfat”)	0.840	1.000													
NIIshare	−0.080	−0.070	1.000												
HHInetvsnon	−0.090	−0.080	0.790	1.000											
Capital	−0.110	−0.150	−0.010	−0.060	1.000										
Liquidity	−0.030	−0.100	0.030	0.030	0.310	1.000									
Size	0.010	0.050	0.030	0.140	−0.720	−0.430	1.000								
Lending rates	0.010	−0.050	0.010	−0.090	0.340	0.450	−0.340	1.000							
Refinancing rates	−0.020	−0.080	−0.060	−0.180	0.230	0.330	−0.220	0.910	1.000						
Rediscounting rates	−0.030	−0.070	−0.070	−0.170	0.220	0.300	−0.200	0.890	0.980	1.000					
Foreign exchange reserves	0.030	0.070	0.130	0.240	−0.060	−0.030	0.070	−0.410	−0.550	−0.540	1.000				
Central bank claims	−0.020	−0.070	−0.080	−0.140	0.010	0.020	−0.030	0.140	0.270	0.210	−0.710	1.000			
Inflation	0.020	−0.020	0.040	−0.030	0.340	0.450	−0.330	0.930	0.860	0.880	−0.250	−0.040	1.000		
Economic growth	−0.130	−0.050	0.050	0.110	−0.230	−0.080	0.220	−0.440	−0.380	−0.340	0.470	−0.160	−0.370	1.000	
Stock return	0.000	−0.010	0.030	0.030	−0.160	−0.180	0.090	−0.550	−0.530	−0.650	0.080	0.040	−0.670	−0.040	1.000

### 4.2 Main results

We report the results obtained from the equations of “cat fat” in Table 4, Table 5 and “cat nonfat” in Table 6, Table 7. We alternatively utilize the variables of income models in each case.

**Table 4:** Non-interest income share and bank liquidity creation (“cat fat”).

	Dependent variable: Liquidity creation (“cat fat”)				
	(1)	(2)	(3)	(4)	(5)
Lagged dependent variable	0.078***	0.088***	0.093***	0.083***	0.096***
	(0.016)	(0.011)	(0.012)	(0.010)	(0.007)
Lending rates	−4.501***				
	(1.415)				
NIIshare*Lending rates	−0.021***				
	(0.004)				
Refinancing rates		−2.817**			
		(1.094)			
NIIshare*Refinancing rates		−0.009***			
		(0.000)			
Rediscounting rates			−3.980***		
			(1.163)		
NIIshare*Rediscounting rates			−0.010***		
			(0.001)		
Foreign exchange reserves				1.804***	
				(0.316)	
NIIshare*Foreign exchange reserves				−0.020***	
				(0.002)	
Central bank claims					6.388***
					(0.955)
NIIshare*Central bank claims					−0.004***
					(0.000)
NIIshare	0.284	0.092	0.081	0.271	0.023
	(0.362)	(0.144)	(0.134)	(0.190)	(0.118)
Size	1.807	3.772**	3.699**	2.277	5.155**
	(3.817)	(1.857)	(1.875)	(2.475)	(2.088)
Capital	−1.186	−0.181	−0.169	−0.746	−0.156
	(0.912)	(0.681)	(0.672)	(0.524)	(0.746)
Liquidity	2.870***	1.986***	2.076***	2.186***	1.693***
	(0.384)	(0.179)	(0.186)	(0.357)	(0.233)
Economic growth	−9.870***	−2.835	−1.602	−11.389***	−6.613***
	(3.069)	(1.785)	(1.788)	(2.315)	(1.910)
Stockgrowth	−0.412***	−0.309***	−0.293***	−0.212***	−0.334***
	(0.067)	(0.043)	(0.043)	(0.044)	(0.041)
Observations	331	331	331	331	331
Banks	31	31	31	31	31
Instruments	29	29	29	29	29
AR (1) test (p-value)	0.011	0.014	0.014	0.013	0.012
AR (2) test (p-value)	0.570	0.639	0.624	0.537	0.717
Hansen test (p-value)	0.250	0.329	0.299	0.537	0.546

The table reports the results using the two-step system GMM estimator. The dependent variable is liquidity creation (“cat fat”), as discussed in subsection 3.3.1. The independent variables of main interest are alternative monetary policy indicators (as discussed in subsection 3.3.2) and their interactions with the non-interest income share (NIIshare). Other controls are presented in Table 2. Standard errors are displayed in parentheses. *** and ** indicate the significance levels at 1% and 5%, respectively.

**Table 5:** Income diversification and bank liquidity creation (“cat fat”).

	Dependent variable: Liquidity creation (“cat fat”)				
	(1)	(2)	(3)	(4)	(5)
Lagged dependent variable	0.128***	0.139***	0.064***	0.030***	0.112***
	(0.013)	(0.008)	(0.012)	(0.010)	(0.006)
Lending rates	−2.533***				
	(0.637)				
HHInetvsnon*Lending rates	−4.969**				
	(2.111)				
Refinancing rates		−1.241*			
		(0.683)			
HHInetvsnon*Refinancing rates		−13.186***			
		(2.611)			
Rediscounting rates			−3.597***		
			(1.055)		
HHInetvsnon*Rediscounting rates			−10.407***		
			(3.671)		
Foreign exchange reserves				2.873***	
				(0.763)	
HHInetvsnon*Foreign exchange reserves				−7.251***	
				(2.653)	
Central bank claims					3.577**
					(1.412)
HHInetvsnon*Central bank claims					−2.098***
					(0.640)
HHInetvsnon	−23.361**	−5.196	−80.142***	78.089	−12.141
	(11.841)	(10.443)	(21.038)	(49.268)	(15.616)
Size	7.653***	6.892***	4.760**	4.668*	7.265***
	(2.422)	(2.358)	(2.188)	(2.382)	(2.139)
Capital	−0.813	−1.219**	0.153	−0.287	−1.498***
	(0.494)	(0.540)	(0.708)	(0.712)	(0.451)
Liquidity	3.179***	3.044***	2.118***	2.173***	2.626***
	(0.228)	(0.201)	(0.294)	(0.296)	(0.160)
Economic growth	−7.868***	−3.556**	0.405	−4.055**	−6.204***
	(1.770)	(1.573)	(1.114)	(1.873)	(1.406)
Stockgrowth	−0.309***	−0.316***	−0.138***	−0.271***	−0.336***
	(0.042)	(0.040)	(0.053)	(0.049)	(0.036)
Observations	331	331	331	331	331
Banks	31	31	31	31	31
Instruments	29	29	29	29	29
AR (1) test (p-value)	0.016	0.016	0.014	0.015	0.016
AR (2) test (p-value)	0.726	0.715	0.495	0.593	0.635
Hansen test (p-value)	0.252	0.361	0.655	0.704	0.542

The table reports the results using the two-step system GMM estimator. The dependent variable is liquidity creation (“cat fat”), as discussed in subsection 3.3.1. The independent variables of main interest are alternative monetary policy indicators (as discussed in subsection 3.3.2) and their interactions with the income diversification (HHInetvsnon). Other controls are presented in Table 2. Standard errors are displayed in parentheses. ***, **, and * indicate the significance levels at 1%, 5%, and 10%, respectively.

**Table 6:** Non-interest income share and bank liquidity creation (“cat nonfat”).

	Dependent variable: Liquidity creation (“cat nonfat”)				
	(1)	(2)	(3)	(4)	(5)
Lagged dependent variable	0.215***	0.196***	0.197***	0.201***	0.184***
	(0.016)	(0.013)	(0.015)	(0.011)	(0.010)
Lending rates	−4.506***				
	(1.259)				
NIIshare*Lending rates	−0.027***				
	(0.005)				
Refinancing rates		−6.530***			
		(0.993)			
NIIshare*Refinancing rates		−0.008***			
		(0.000)			
Rediscounting rates			−5.929***		
			(0.967)		
NIIshare*Rediscounting rates			−0.009***		
			(0.001)		
Foreign exchange reserves				1.820***	
				(0.260)	
NIIshare*Foreign exchange reserves				−0.014***	
				(0.001)	
Central bank claims					8.304***
					(0.732)
NIIshare*Central bank claims					−0.003***
					(0.000)
NIIshare	0.365	−0.183**	−0.164*	−0.257***	−0.169***
	(0.312)	(0.092)	(0.085)	(0.068)	(0.051)
Size	6.105**	−1.955	−1.970	−0.709	0.356
	(3.095)	(1.675)	(1.538)	(1.082)	(1.124)
Capital	−2.799**	−1.038*	−1.090*	−1.306***	−1.257***
	(1.112)	(0.631)	(0.604)	(0.407)	(0.294)
Liquidity	1.706***	0.945***	0.849***	0.514***	0.003
	(0.223)	(0.140)	(0.140)	(0.173)	(0.173)
Economic growth	−8.447***	−0.764	−0.644	−7.708***	−9.635***
	(1.058)	(1.127)	(1.116)	(1.182)	(1.262)
Stockgrowth	−0.281***	−0.263***	−0.213***	−0.246***	−0.294***
	(0.038)	(0.031)	(0.027)	(0.022)	(0.019)
Observations	331	331	331	331	331
Banks	31	31	31	31	31
Instruments	29	29	29	29	29
AR (1) test (p-value)	0.013	0.024	0.023	0.016	0.017
AR (2) test (p-value)	0.512	0.897	0.897	0.868	0.958
Hansen test (p-value)	0.218	0.126	0.123	0.195	0.142

The table reports the results using the two-step system GMM estimator. The dependent variable is liquidity creation (“cat nonfat”), as discussed in subsection 3.3.1. The independent variables of main interest are alternative monetary policy indicators (as discussed in subsection 3.3.2) and their interactions with the non-interest income share (NIIshare). Other controls are presented in Table 2. Standard errors are displayed in parentheses. ***, **, and * indicate the significance levels at 1%, 5%, and 10%, respectively.

**Table 7:** Income diversification and bank liquidity creation (“cat nonfat”).

	Dependent variable: Liquidity creation (“cat nonfat”)				
	(1)	(2)	(3)	(4)	(5)
Lagged dependent variable	0.297***	0.325***	0.315***	0.150***	0.266***
	(0.022)	(0.019)	(0.021)	(0.011)	(0.009)
Lending rates	−4.081***				
	(1.106)				
HHInetvsnon*Lending rates	−7.563***				
	(2.362)				
Refinancing rates		−3.295**			
		(1.390)			
HHInetvsnon*Refinancing rates		−10.259***			
		(3.204)			
Rediscounting rates			−8.122***		
			(0.976)		
HHInetvsnon*Rediscounting rates			−14.351***		
			(3.687)		
Foreign exchange reserves				3.994***	
				(0.870)	
HHInetvsnon*Foreign exchange reserves				−7.746***	
				(2.766)	
Central bank claims					3.813***
					(1.289)
HHInetvsnon*Central bank claims					−1.145***
					(0.408)
HHInetvsnon	−26.028*	−25.758*	−79.212***	−92.771**	−5.810
	(15.047)	(15.061)	(18.865)	(43.832)	(12.770)
Size	3.912	1.954	2.042	−0.403	2.457**
	(2.484)	(2.383)	(1.632)	(1.384)	(1.006)
Capital	−0.324	−0.609	−0.306	−0.999	−1.049***
	(0.715)	(0.668)	(0.619)	(0.618)	(0.389)
Liquidity	2.069***	1.658***	2.066***	0.381**	0.999***
	(0.219)	(0.202)	(0.180)	(0.168)	(0.113)
Economic growth	−7.516***	−0.884	−0.403	−7.018***	−6.812***
	(1.562)	(1.583)	(1.748)	(1.513)	(1.214)
Stockgrowth	−0.160***	−0.194***	−0.366***	−0.243***	−0.249***
	(0.033)	(0.039)	(0.058)	(0.027)	(0.032)
Observations	331	331	331	331	331
Banks	31	31	31	31	31
Instruments	29	29	29	29	29
AR (1) test (p-value)	0.013	0.014	0.009	0.014	0.011
AR (2) test (p-value)	0.868	0.784	0.972	0.988	0.972
Hansen test (p-value)	0.203	0.206	0.139	0.190	0.122

The table reports the results using the two-step system GMM estimator. The dependent variable is liquidity creation (“cat nonfat”), as discussed in subsection 3.3.1. The independent variables of main interest are alternative monetary policy indicators (as discussed in subsection 3.3.2) and their interactions with the income diversification (HHInetvsnon). Other controls are presented in Table 2. Standard errors are displayed in parentheses. ***, **, and * indicate the significance levels at 1%, 5%, and 10%, respectively.

Looking into the standalone monetary policy indicators, we observe that the coefficients for different interest-rate variables are significantly negative. However, those for quantitative-based instruments, such as open market transactions and foreign currency reserves, are significantly positive. The results remain identical across two versions of bank liquidity measures. Our results are also economically significant. Taking the coefficient in column 2 of Table 4 as an example, a decrease of one percentage point in refinancing rates may yield an increase of 2.817% points in the total liquidity creation growth; similarly, the coefficient in column 5 of Table 6 reveals that a rise of one percentage point in central bank claims causes an increase of 8.304% points in the on-balance sheet liquidity creation expansion. These amounts are moderate since Vietnam's interest framework has always been far away from the zero bound. This argument is more conspicuous if we compare our findings with those of Berger and Bouwman [7]. The prior authors reveal that a decrease of one percentage point in the federal funds rate (a tremendous change in monetary policy) might boost liquidity creation (only at small banks) by 2.0–2.3% points. The magnitudes of the impact slightly change in other columns but still highlight the economic plausibility. Overall, we highly confirm the existence of the bank liquidity creation channel proposed by Berger and Bouwman [7].

We next focus on the interaction terms of primary interest. The regressions with all interest rates show that the interaction terms between monetary policy and strategic scope proxies are significantly negative. Considering the highly negative coefficient on the isolated monetary policy indicators that we computed before, these results allow us to argue that bank liquidity creation is more sensitive to monetary interest rate shocks if banks’ business models exhibit a larger non-interest income ratio or a higher degree of diversification. This finding is economically significant. For instance, a one standard deviation increase in the diversity degree may amplify the impact of a one percentage point change in lending rates on the total liquidity growth by approximately 0.621% points (0.125 × 4.969, column 1 of Table 5). The economic significance is also observed in other remaining regressions.

Turning to the analysis of two quantitative-based indicators, we observe the negative and statistically significant coefficients on the interaction terms between bank income and monetary policy, captured by both the SBV's securities trading and foreign exchange holdings. As displayed in all tables, our regression pattern is robust across two different measures of bank liquidity creation. Regarding the economic significance, for example, we calculate that an increase of one standard deviation in the non-interest income ratio should reduce the effect of a one percentage point change in foreign exchange reserves on the on-balance sheet liquidity creation growth by about 0.209% points (14.907 × 0.014, column 4 of Table 6). Other remaining regressions still highlight the economic plausibility of our findings. All in all, contradicting coefficient signs between monetary indicators and interaction terms are interpreted as evidence that the liquidity creation channel is mitigated at diversified banks with more reliance on non-interest income. This event occurs when the central bank alters the money injection in the market.

The empirical findings with interest- and quantitative-based monetary tools provide contradictory evidence with respect to the marginal effects of strategic scope on the monetary policy transmission via the bank liquidity creation channel. More precisely, findings from three types of interest rates imply that non-interest income reliance/income diversification nourishes monetary policy transmission potency. However, results from the SBV's securities trading and foreign exchange holdings signify that a higher level of income diversification or a more dependence on non-interest income undermines the extent that bank liquidity reacts to monetary adjustments. Our work expands the literature stream engaged by previous authors [9,10,12], who explore how standard bank balance sheet characteristics drive the bank creation channel. Some potential mechanisms to explain our findings could be as follows. First, banks with more non-interest-based activities may have lower switching costs, leading to more unstable client relationships [17]. So, when interest rates are adjusted, the activities of these banks will be more affected. This route constitutes the strengthening effects of income models. Second, as a pure fact, banks with higher income diversification may prefer to place their funds in different investment categories other than loan holdings. Thus, the central bank pours additional cash into the financial system, banks may have other investment opportunities to pursue (such as securities trading) rather than creating liquidity for the public. This manner forms the weakening impacts of business models.^7^

We should also discuss the results for our control variables. Many interesting significant findings have appeared in this regard. The coefficients on the non-interest income share and the income diversification measure are significantly negative. These results are in line with many previous papers which find that bank diversification negatively affects liquidity formation [31,32]. We detect positive coefficients for bank size, implying that larger banks may have higher liquidity creation growth. This result is probably owing to the hypothesis of the scale economies that benefits larger banks [44]. The coefficient on bank capital is negative and statistically significant, indicating that a larger buffer of capital is associated with less liquidity creation expansion, consistent with the “financial fragility” hypothesis [45]. The proportion of liquid assets is significantly positive, supporting the view that liquid banks may extend their core function subsequently, concurring with the argument of [47]. Finally, we find that bank liquidity creation tends to strengthen when the economy and the stock market slow down, evidenced by the negative coefficients on the GDP growth and the stock market. These findings contrast with those obtained in prior studies [13,48]. Due to the precautionary motive when banks predict that they cannot address momentary liquidity crises during economic booms [49], they may stockpile a more fabulous liquidity cushion instead of releasing extra liquidity.

### 4.3 Robustness tests

We now carry out some robustness estimates to examine the sensitivity of the findings presented earlier in the paper. To this end, using a different income model measure and an alternative econometric methodology would be considered. We first replace the measure capturing the income diversification between traditional and non-traditional activities with a new measure of income diversification across all bank activities. Inspired by Hou et al. [31] and Shim [18], this new income diversification indicator is calculated based on equation [5] as follows:

(5) $$HHIall=1\;–{\sum_{i=1}^{5}\;\left(\frac{I{ncome}_{i}}{Operating\;income}\right)}^{2}$$

where ${Income}_{i}$ is the income source i and each Vietnamese bank's operating income contains five main categories: net interest income, service revenue, trading income in foreign currencies, investment income from securities, and other non-interest income. Moreover, our original sample from 2007 to 2019 may suffer from a significant structural break due to the global financial crisis, which may substantially distort the monetary policy transmission. Therefore, we adjust our present sample by excluding the years 2007–2009 affected by crisis shocks. The subsample results with the new income model measure, using the GMM estimator, are displayed in Table 8.

**Table 8:** Subsample checks with an alternative measure of income diversification.

	Dependent variable: Liquidity creation (“cat fat”)					Dependent variable: Liquidity creation (“cat nonfat”)				
	(1)	(2)	(3)	(4)	(5)	(6)	(7)	(8)	(9)	(10)
Lagged dependent variable	0.139***	0.140***	0.099***	0.133***	0.055***	0.309***	0.319***	0.267***	0.306***	0.156***
	(0.013)	(0.010)	(0.011)	(0.017)	(0.007)	(0.022)	(0.013)	(0.018)	(0.009)	(0.008)
Lending rates	−1.885**					−3.643***				
	(0.892)					(1.290)				
HHIall*Lending rates	−9.703***					−6.520***				
	(2.245)					(1.860)				
Refinancing rates		−2.574***					−1.737**			
		(0.808)					(0.877)			
HHIall*Refinancing rates		−11.559***					−10.611***			
		(1.575)					(1.175)			
Rediscounting rates			−5.254***					−3.684***		
			(0.800)					(1.132)		
HHIall*Rediscounting rates			−18.863***					−22.856***		
			(6.341)					(2.847)		
Foreign exchange reserves				3.886***					2.540***	
				(0.974)					(0.556)	
HHIall*Foreign exchange reserves				−5.990***					−3.632***	
				(1.622)					(1.108)	
Central bank claims					2.513***					4.674***
					(0.887)					(1.252)
HHIall*Central bank claims					−7.900***					−8.511***
					(2.941)					(2.758)
HHIall	−23.395***	−7.349	−56.060*	−2.890	−20.794**	9.986	5.646	−89.592***	4.060	−26.321***
	(6.882)	(8.171)	(32.202)	(14.133)	(8.795)	(12.286)	(9.785)	(15.685)	(13.336)	(8.526)
***Controls***	***Yes***	***Yes***	***Yes***	***Yes***	***Yes***	***Yes***	***Yes***	***Yes***	***Yes***	***Yes***
Observations	274	274	274	274	274	274	274	274	274	274
Banks	31	31	31	31	31	31	31	31	31	31
Instruments	29	29	29	29	29	29	29	29	29	29
AR (1) test (p-value)	0.021	0.021	0.021	0.021	0.013	0.020	0.023	0.007	0.018	0.017
AR (2) test (p-value)	0.895	0.752	0.594	0.605	0.540	0.911	0.842	0.903	0.992	0.787
Hansen test (p-value)	0.268	0.610	0.468	0.315	0.399	0.260	0.302	0.134	0.338	0.116

The table reports the results using the two-step system GMM estimator. The dependent variables are total liquidity creation (“cat fat”, columns 1–5) and on-balance-sheet liquidity creation (“cat nonfat”, columns 6–10), as discussed in subsection 3.3.1. The independent variables of main interest are alternative monetary policy indicators (as discussed in subsection 3.3.2) and their interactions with alternative income diversification (HHIall). Other controls are presented in Table 2. The table reports the results based on a subsample from 2010 to 2019. Standard errors are displayed in parentheses. ***, **, and * indicate the significance levels at 1%, 5%, and 10%, respectively.

Second, we modify our initial model specification and then apply a different econometric methodology. In more detail, we drop the lagged dependent variable in the dynamic equation to adopt the static model. To create efficient estimates, we follow Hoechle [50]'s regression procedure, which produces fixed-effects regression with Driscoll-Kraay standard errors. These standard errors accordingly are heteroscedasticity and autocorrelation consistent and robust to the cross-sectional dependence. We repeat the regressions with the new econometric methodology and report the results in Table 9.^8^

**Table 9:** Robustness checks with an alternative empirical approach.

	Dependent variable: Liquidity creation (“cat fat”)					Dependent variable: Liquidity creation (“cat nonfat”)				
	(1)	(2)	(3)	(4)	(5)	(6)	(7)	(8)	(9)	(10)
Lending rates	−4.352***					−5.283***				
	(0.877)					(0.435)				
NIIshare*Lending rates	−0.010***					−0.010**				
	(0.003)					(0.004)				
Refinancing rates		−6.032***					−6.435***			
		(0.682)					(0.734)			
NIIshare*Refinancing rates		−0.012***					−0.012**			
		(0.003)					(0.004)			
Rediscounting rates			−5.845***					−5.936***		
			(0.665)					(0.710)		
NIIshare*Rediscounting rates			−0.014***					−0.014**		
			(0.004)					(0.005)		
Foreign exchange reserves				2.924*					2.902**	
				(1.600)					(1.278)	
NIIshare*Foreign exchange reserves				−0.022***					−0.019**	
				(0.007)					(0.007)	
Central bank claims					17.773***					11.672***
					(4.453)					(3.077)
NIIshare*Central bank claims					−0.007***					−0.007***
					(0.001)					(0.001)
NIIshare	−0.559*	−0.595**	−0.595**	−0.342	−0.244	−0.377**	−0.402**	−0.397**	−0.200	−0.234*
	(0.254)	(0.261)	(0.261)	(0.315)	(0.215)	(0.156)	(0.159)	(0.158)	(0.203)	(0.111)
***Controls***	***Yes***	***Yes***	***Yes***	***Yes***	***Yes***	***Yes***	***Yes***	***Yes***	***Yes***	***Yes***
Observations	331	331	331	331	331	331	331	331	331	331
Banks	31	31	31	31	31	31	31	31	31	31
F-test (p-value)	0.000	0.000	0.000	0.000	0.000	0.000	0.000	0.000	0.000	0.000
R-squared	0.164	0.172	0.171	0.158	0.142	0.103	0.107	0.102	0.083	0.105

The table reports the results of the fixed-effects regressions with Driscoll-Kraay standard errors in static panel models. The dependent variables are total liquidity creation (“cat fat”, columns 1–5) and on-balance-sheet liquidity creation (“cat nonfat”, columns 6–10), as discussed in subsection 3.3.1. The independent variables of main interest are alternative monetary policy indicators (as discussed in subsection 3.3.2) and their interactions with alternative income diversification (HHIall). Other controls are presented in Table 2. Standard errors are displayed in parentheses. ***, **, and * indicate the significance levels at 1%, 5%, and 10%, respectively.

Taken together, the results still suggest that bank liquidity creation improves as interest rates decrease or money supply increases, consistent with the proposition of the liquidity creation channel. Importantly, for income models that rely more on non-interest sources or highlight higher bank diversification, we also find that the influence of monetary policy on liquidity creation is more pronounced when monetary policy is conducted via interest rate changes but less conspicuous if the central bank modifies its liquidity injection.

## 5 Conclusions

The paper analyzes the marginal impact of strategic scope on the effects of monetary policy on liquidity creation growth. In contrast to the rich literature that widely exhibits the existence and working of the bank lending channel, the bank liquidity creation channel needs to be explored further. Using a sample of Vietnamese commercial banks over the period from 2007 to 2019, we find multiple interesting results that could be summarized as follows. We document that the bank liquidity creation channel operates in Vietnam. Concretely, banks respond to an expansionary policy, either when the SBV reduces its policy rates or injects more money into the economy, by increasing the liquidity creation ability to a larger extent. Besides, our key contribution in this paper shows that the use of multiple different monetary policy measures allows us to argue that the implications regarding the moderating role of strategic scope could be misleading if based on a single monetary indicator. At banks with income models that rely more on non-interest income or diversify more into various banking segments, the linkage between monetary policy and bank liquidity creation is strengthened if the SBV adjusts interest rates but mitigated when it changes the money supply using quantitative-based monetary tools.

Accordingly, we propose careful surveillance of diversification strategies that shape banks' strategic scope, which significantly plays an important conditioning role in how bank liquidity creation responds to monetary shifts. Central banks should provide targeted guidance for banks’ business models because of the complicated asymmetric link between income diversification and the bank liquidity creation channel. Also, in light of our findings, central banks in multiple-tool environments should take into consideration the different transmission mechanisms between quantitative- and interest-based tools when implementing their monetary policy. From a research perspective, we admit that the liquidity creation channel broadens the lending channel of monetary policy, thus opening up a new and interesting avenue for further research.

We admit that the small sample size in a single country is a limitation of this study, which may also restrict the generalizability of the findings. We recommend that more research should be conducted in the future with other individual markets and/or cross-country designs in order to expand the body of knowledge on this current subject.

## Data availability statement

Data will be made available on request.

## Funding

This research received no specific funding.

## Additional information

No additional information is available for this paper.

## CRediT authorship contribution statement

**Japan Huynh:** Writing - review & editing, Writing - original draft, Visualization, Validation, Software, Investigation, Formal analysis, Data curation, Conceptualization.

## Declaration of competing interest

The authors declare that they have no known competing financial interests or personal relationships that could have appeared to influence the work reported in this paper.

## Footnotes

1 For an excellent review of the literature stream on the determinants of bank liquidity creation, please refer to Ali et al. [51], Evans and Haq [52], and Guo and Zhang [53].

2 This filtering is crucial in examining the second-order autocorrelation, which we carry out to ensure the consistency of the estimation results obtained by the GMM estimator [54].

3 In a pure floating exchange rate system, the exchange rate is established by the equilibrium between private market demand and private market supply for a currency. The central bank is not inherently involved in determining a pure floating exchange rate. This implies that the central bank does not intervene by buying and selling foreign currency, or in other words, does not change foreign exchange reserves. Hence, the standard deviation of the foreign exchange reserves *[σ(fxr)]* should have the value of 0.

4 With the purely fixed exchange rate system scenario, it can be simply understood that there is no fluctuation in the effective exchange rate. Thus, the standard deviation of the effective exchange rate *[σ(eer)]* should have the value of 0.

5 The detailed definitions of all control variables are shown in Table 2.

6 Concerning the issue of multicollinearity, we observe that the explanatory variables are not significantly correlated with one another, with the exception of the high correlation between bank size and capital (−0.720). Nevertheless, we are not concerned because (i) correlations between variables exceeding 0.80 do indeed lead to severe multicollinearity [55]; (ii) additional checks utilizing the variance inflation factor (VIF) show that the VIF value is less than 10 and our VIF results confirm the notion that our model is safely free from the serious multicollinearity problem when we leave the inflation variable out of the model; (iii) the GMM estimator is robust to the multicollinearity problem [56]; and (iv) these two variables are not the primary focus of our study. Multiple arguments corroborate that our model is free of the severe multicollinearity issue. So, we do not present the VIF results for the sake of brevity (this is only one of the reasons supporting our decision), given the fact that our paper already contained up to nine tables of results.

7 Our explanation should be accompanied by a caveat stating that, in reality, the Vietnamese government has enacted regulations restricting bank purchases of securities. For example, banks can only invest in sovereign bonds using a maximum of 35% short-term funding. This might diminish our potential channels for explaining the current pattern. We thank an anonymous referee for this point.

8 We report the results using the non-interest income share in Table 9. For those obtained using income diversification, they still offer identical conclusions but are omitted for the sake of brevity. Besides, analysis with the alternative econometric for the subsample without the financial crisis period also yields the same outcomes. All of these results are always available upon request.
